# Association of LDL protein cargo with segmental pulse wave velocity in older adults in the atherosclerosis risk in communities study

**DOI:** 10.3389/fragi.2026.1691150

**Published:** 2026-05-22

**Authors:** Binchong An, Pamela L. Lutsey, Michelle M. Mielke, Fang Yu, Ron C. Hoogeveen, Lin Zhang, Alvaro Alonso, Michelle Meyer, Danni Li

**Affiliations:** 1 Department of Lab Medicine Pathology, University of Minnesota, Minneapolis, MN, United States; 2 Division of Epidemiology and Community Health, School of Public Health, University of Minnesota, Minneapolis, MN, United States; 3 Department of Epidemiology and Prevention, Wake Forest University School of Medicine, Winston-Salem, NC, United States; 4 Edson College of Nursing and Health Innovation, Arizona State University, Phoenix, AZ, United States; 5 Division of Cardiovascular Research, Department of Medicine, Baylor College of Medicine, Houston, TX, United States; 6 Division of Biostatistics and Health Data Science, School of Public Health, University of Minnesota, Minneapolis, MN, United States; 7 Department of Epidemiology, Rollins School of Public Health, Emory University, Atlanta, GA, United States; 8 Gillings School of Global Public Health, Department of Epidemiology, University of North Carolina at Chapel Hill, Chapel Hill, NC, United States

**Keywords:** apolipoprotein B, arterial stiffness, baPWV, cfPWV, complement component C3, LDL

## Abstract

**Background:**

Low-density lipoprotein cholesterol (LDL-C) is associated with brachial-ankle pulse wave velocity (baPWV). However, its relationship with femoral-ankle PWV (faPWV) or carotid-femoral PWV (cfPWV) is less consistent. In addition to its lipid cargo, LDL also has protein cargo. This study aims to examine and compare cross-sectional associations between LDL protein cargo or LDL-C and segment-specific PWV measures in an aging population.

**Methods:**

The study included 65 participants without dementia from the Atherosclerosis Risk in Communities (ARIC) study, with LDL separated from their plasma. LDL cargo proteins were quantified using a proteomic method based on mass spectrometry. Linear regression was utilized to evaluate the cross-sectional associations between LDL cargo protein or LDL-C and baPWV, faPWV, or cfPWV, after adjusting for covariates.

**Results:**

The 65 participants had a mean age of 76.3 years (SD = 5.4), 53.8% (35/65) were female, 30.8% (20/65) were Black. In the fully adjusted models, each SD increment of apolipoprotein B (apoB) in LDL and LDL-C was positively associated with an increase in baPWV of 103.0 cm/s (95% CI [43.3, 162.6]; p = 0.001) and 54.3 cm/s (95% CI [−5.9, 114.6]; p = 0.08), respectively. Each SD increment of complement component 3 (C3) in LDL was positively associated with an increase in cfPWV of 80.9 cm/s (95% CI [14.0, 147.7]; p = 0.02), but each SD increment of LDL-C was negatively associated with a decrease in cfPWV of 62.4 cm/s (95% CI [−123.1, −1.8]; p = 0.04).

**Conclusion:**

We identified differential associations of LDL cargo protein apoB and C3 as well as LDL-C with baPWV or cfPWV.

## Introduction

Low-density lipoprotein cholesterol (LDL-C), a primary lipid cargo carried by plasma LDL, is recognized as a key determinant of risk and a therapeutic target in atherosclerotic cardiovascular disease (Grundy et al., 2018). LDL-C is associated with brachial-ankle PWV (baPWV) (Zhao et al., 2014; Liu et al., 2022), an integrative measure of stiffness across central and peripheral arteries (Stone et al., 2023). baPWV adds independent predictive values to the traditional biomarkers for cardiovascular disease (Stone et al., 2023). However, the relationships between LDL-C and other segment-specific PWV measures, such as femoral-ankle PWV (faPWV) and carotid-femoral (cfPWV), which measure peripheral and central arterial stiffness, respectively, are less consistent (Heffernan et al., 2022; Choo et al., 2014; Vishnu et al., 2014). Peripheral and central arterial territories have very different structural components, in that, peripheral arteries are richer in collagen fibers and vascular smooth muscle cells, and central arteries have a higher proportion of elastin (Jadidi et al., 2021). The differential associations of LDL-C with segment-specific PWVs are likely due to the differences in arterial architecture and functions across the arterial segments, as well as the distinct patterns of arterial tissue remodeling related to aging and vascular risk factors across different arterial territories (Meyer et al., 2016a).

Apolipoprotein B (apoB) is a major protein of LDL. Plasma apoB, which measures mainly apoB in LDL, despite other apoB-containing lipoproteins such as very-low-density lipoprotein (VLDL), has also been consistently associated with baPWV (Qu et al., 2021). In addition to apoB, plasma LDL contains other protein cargo (Dashty et al., 2014). However, no studies have investigated the associations between protein cargo, including apoB within LDL, and PWVs. Studies of LDL protein cargo require isolation of LDL from plasma. In recent years, an anion exchange fast protein liquid chromatography (AEX-FPLC) method has been established in our lab to isolate plasma lipoproteins, including LDL. Furthermore, a label-free quantitative mass spectrometry technique has been developed to identify proteins along with their relative abundances in fractionated lipoproteins. Applying these new methodologies, we examined the associations between LDL cargo proteins with segment-specific PWV (baPWV, faPWV, cfPWV) in the aging population in the Atherosclerosis Risk in Communities (ARIC) study. Additionally, we compared the associations between LDL cargo proteins and these PWVs to those of LDL-C.

## Methods

### Study population

The ARIC study was a prospective epidemiological investigation conducted across four U.S. communities, originally designed to investigate the causes of atherosclerosis and cardiovascular disease. A detailed description of the study design was published (Wright et al., 2021). The ARIC-NCS study was an ancillary study of ARIC that examined midlife risk factors for dementia (Knopman et al., 2016). Between 2011 and 2013, 6,538 participants attended Visit 5. As part of the study protocol, participants completed a physical examination and a comprehensive neurocognitive assessment. A subset of participants also underwent a brain MRI scan. Concurrently, blood and urine specimens were collected, processed, and stored for future analysis. For this particular study, we used the following inclusion criteria to select eligible ARIC-NCS participants: 1) the availability of “frozen never-thawed” fasting plasma samples at the ARIC-NCS baseline; 2) cognitive status was adjudicated as either cognitively normal (CN) or mild cognitive impairment (MCI) at the ARIC-NCS baseline; 3) availability of brain MRI scans at the ARIC-NCS baseline; and 4) availability of PET scans with ^18^F-AV-45 (florbetapir) in the ARIC-PET study in 2012–15 (baseline). Eventually, we randomly selected 65 ARIC participants from the 340 participants meeting these eligibility criteria.

### Assessment of the relative abundance of proteins in fractionated LDL

We used the same protocols detailed previously to isolate plasma lipoproteins, including LDL, and to identify and quantify LDL cargo proteins (Li et al., 2025). Missing values were assumed to be missing at random and were handled using multiple imputations by chained equations (MICE), employing a random-forest algorithm for estimation (Van Buuren and Groothuis-Oudshoorn, 2011). 89 LDL cargo proteins were identified, with 28 overlapping with proteins reported in four prior studies in the LDL proteome database. Given the strong consensus regarding these 28 proteins as LDL cargo proteins, our analyses are focused exclusively on this subset.

### Segment-specific PWV measures

After resting in a supine position for 5–10 min, the segment-specific PWVs of participants were measured by trained technicians using an automated waveform analyzer VP-1000 Plus (Omron Co., Ltd., Kyoto, Japan), under a standardized protocol (Meyer et al., 2016a). Generally, PWV was calculated by dividing the distance between two arterial recording sites by travel time. The distance for cfPWV estimation was directly obtained using a segmometer designed for PWV measurements (Rosscraft, Surrey, Canada). It was calculated as the distance from the carotid to the femoral, subtracting the distance from the suprasternal notch to the carotid. In contrast, the VP-1000 Plus, which utilizes height-based equations, was used to calculate distances for baPWV and faPWV automatically (Tanaka et al., 2009).

To calculate the travel time, applanation tonometry sensors were utilized to capture the waveforms of the carotid and femoral arteries simultaneously over a 30-s period, with sensors attached to the left common carotid artery and left common femoral artery. A plethysmographic and an oscillometric pressure sensor attached to cuffs were utilized to capture the waveforms of the bilateral brachial and posterior tibial arteries over a 10-s period, with cuffs wrapped on both arms and ankles, respectively. According to the protocol, each participant was measured at least twice, with the average of the last two measurements. Quality assurance procedures were conducted by one of the authors (H.T.) on center-stratified random samples of 40 records per month, with feedback provided to the technicians (Meyer et al., 2016b). These procedures included centralized training and recertification, quarterly equipment calibration, and ongoing quality control reviews. We included the right baPWV, right faPWV, and cfPWV measurements for this analysis. Measurement reliability, assessed by intra-class correlation coefficients and 95% confidence intervals, was 0.84 (95% CI: 0.78–0.90) for baPWV, 0.69 (95% CI: 0.59–0.79) for faPWV, and 0.70 (95% CI: 0.59–0.81) for cfPWV (Meyer et al., 2016b).

### LDL-C and covariates

ARIC Visit 5 collected blood samples following a fasting period of at least 12 h. Plasma total cholesterol and triglycerides were measured using enzymatic methods, and high-density lipoprotein cholesterol (HDL-C) was measured using an enzymatic method after precipitation of plasma using dextran-magnesium. Low-density lipoprotein cholesterol (LDL-C) was estimated based on the Friedewald formula: $\text{LDL}‐C=Total\;Cholesterol-HDL‐C-\frac{Triglycerides}{5}$ (Zhang et al., 2023). At ARIC visit 5, as part of the ARIC-NCS, participants underwent a comprehensive neurocognitive battery and a neurological examination. Additionally, a subset of participants also had a brain MRI. As previously described, cases of mild cognitive impairment (MCI) and dementia were adjudicated by an expert committee based on the collected information during the ARIC-NCS and the results from prior cognitive testing (Knopman et al., 2016). Information on participants’ education level, gender, race, smoking status, and medication use was self-reported at the study baseline or all study visits. Body mass index (BMI) was calculated using the following equation: $BMI=\frac{weight\;\left(kg\right)}{{\left[\text{height}\;\left(m\right)\right]}^{2}}$ . Prevalent coronary heart disease, heart failure, and stroke were determined based on established criteria published in prior literature (Loehr et al., 2008; Rosamond et al., 1999; Folsom et al., 2002). Diabetes was considered prevalent if participants reported a physician diagnosis of diabetes, were using antidiabetic medications, fasting blood glucose of ≥126 mg/dL, or had an HbA_1c_ level of 6.5% or higher measured during ARIC visit 5. *APOE4* genotype was identified using the TaqMan SNP Genotyping assay (Applied Biosystems, Foster City, CA) (Knopman et al., 2009). Mean Arterial Pressure (MAP) was calculated using diastolic blood pressure (DBP) and systolic blood pressure (SBP) according to the equation: $MAP=DBP+\frac{\left(SBP-DBP\right)}{3}$ (Van Popele et al., 2001).

### Statistical analysis

Before conducting analysis, any protein measured in the LDL fraction with greater than 40% missing values across all samples was excluded. Among the 89 retained for analysis, the proportion of missing values ranged up to 30.77%, with an average of 3.98%, in line with the rates observed in other data-independent acquisition (DIA) proteomics studies (Muntel et al., 2019). Additionally, any protein that was not present in LDL Proteome Watch (last updated 18 February 2015), curated by Dr. Sean Davidson’s laboratory (https://homepages.uc.edu/∼davidswm/LDLproteome.html), was excluded, leaving 28 LDL cargo proteins for data analysis.

Missing protein values were imputed using multiple imputations by chained equations (MICE). The multiple imputation procedure involved creating 40 imputed datasets, with 30 iterations conducted for each dataset to ensure model convergence and stable parameter estimates. Cross-sectional associations between log_2_-transformed LDL cargo protein or LDL-C(mg/dL) and pulse wave velocity (PWV) were examined using linear regression. A minimal set of 9 covariates was included in our analysis: age, sex (male or female), race (white persons or black persons), education levels (basic: less than high school, intermediate: high school or equivalent, high: at least some college), *APOE4* status (yes, no or missing), diabetes(yes or no), cognitive status (cognitive normal or mild cognitive impairment (MCI)), hypertension (yes or no), and mean arterial pressure. Model 1 included no covariate adjustment. Model 2 was adjusted for 6 covariates, including age, sex, race, *APOE4* status, education, and cognitive status. Model 3 included full adjustment for all nine covariates. The two-sided p-value threshold for statistical significance is less than 0.05. We did not adjust for multiple comparisons (i.e., unadjusted p-values) due to the study’s exploratory nature and the small sample size. However, we included adjusted p-values for multiple comparisons using the Benjamini–Hochberg (BH) method in Supplementary Tables. To compare the estimated effect size of LDL cargo proteins to LDL-C, we standardized the beta coefficients of linear regression.

## Results

### Study participants characteristics

This study comprised 65 participants without dementia from the ARIC cohort. The mean age was 76.3 years (SD: 5.4), with 54% (35/65) being female and 31% (20/65) being Blank. Among the 65 participants, 72% (47/65) were adjudicated as cognitively normal (CN), while 28% (18/65) had mild cognitive impairment (MCI). Detailed participant characteristics are presented in Table 1. Notably, the demographic and clinical characteristics of the study participants did not differ significantly from those of eligible individuals who were not selected for the study (see Supplementary Table 1).

**TABLE 1:** Characteristics of the study participants.

Variables	Count (%) or mean (SD)
N	65
Age (year)	76.3 (5.4)
Sex	
Female (n [%])	35 (54%)
Male (n [%])	30 (46%)
Race	
Black (n [%])	20 (31%)
White (n [%])	45 (69%)
BMI (kg/m^2^)	29.7 (5.2)
Cognitive status diagnosis	
Normal (n [%])	47 (71%)
MCI (n [%])	18 (28%)
*APOE4* carrier status	
No (n [%])	47 (72%)
Yes (n [%])	18 (28%)
Missing (n [%])	1(2%)
Education level	
Basic-less than completed high school (n [%])	13 (20%)
Intermediate-completed high school or equivalent (n [%])	26 (40%)
High-completed at least some college (n [%])	26 (40%)
ARIC field center	
Forsyth county, NC (n [%])	19 (29%)
Jackson city, MS (n [%])	19 (29%)
Washington county, MD (n [%])	27 (42%)
Current cigarette smoking status	
Non-smoker (n [%])	59 (91%)
Smoker (n [%])	5 (8%)
Missing (n [%])	1 (2%)
Diabetes prevalence	
No (n [%])	43 (66%)
Yes (n [%])	21 (32%)
Missing (n [%])	1 (2%)
Hypertension prevalence	
No (n [%])	20 (31%)
Yes (n [%])	45 (69%)
Coronary heart disease prevalence	
No (n [%])	56 (86%)
Yes (n [%])	9 (14%)
Stroke prevalence	
No (n [%])	63 (97%)
Yes (n [%])	2 (3%)
Definite or possible heart failure prevalence	
No (n [%])	58 (89%)
Yes (n [%])	7 (11%)
Cholesterol lowering medication use	
No (n [%])	31 (48%)
Yes (n [%])	34 (52%)
Antihypertensive medication use	
No (n [%])	13 (20%)
Yes (n [%])	52 (80%)
Statin use	
No (n [%])	32 (49%)
Yes (n [%])	33 (51%)
HDL-cholesterol (mg/dL)	48.8 (11.6)
LDL-cholesterol (mg/dL)	103.8 (36.4)
Total cholesterol (mg/dL)	179.2 (43.7)
Triglycerides (mg/dL)	133.2 (54.0)
Systolic blood pressure (mmHg)	126.8 (11.3)
Diastolic blood pressure (mmHg)	64.8 (10.3)
cfPWV(cm/s)	1093.4 (264.4)
baPWV(cm/s)	1674.6 (265.2)
faPWV(cm/s)	1075.9 (188.2)
Mean arterial pressure (mmHg)	85.4 (9.3)

### LDL cargo proteins

We identified 89 LDL cargo proteins, 28 of which overlapped with proteins identified by four previous studies in the LDL proteome database. Main biological processes of these 28 LDL cargo proteins are lipid transportation and lipid binding, although a few proteins are involved in complement activation and coagulation such as complement component 3 (C3) and fibrinogen subunits. Some LDL cargo proteins were strongly correlated with LDL-C such as apolipoprotein E, apolipoprotein C4, apolipoprotein M, apolipoprotein L1 (apoL1), apolipoprotein B (apoB), although the majority of them were not correlated with LDL-C. Supplementary Tables 2A,B list the mean log-2 intensity of the 28 LDL proteins and their Pearson correlation coefficients with LDL-C.

### Associations between LDL protein cargo or LDL-C and baPWV

**FIGURE 1:**
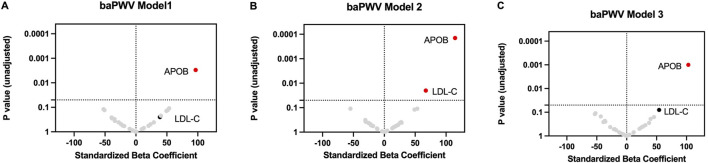
Association of LDL cargo proteins and LDL-C with baPWV. Linear regression was used to assess the associations between each LDL cargo protein (log_2_-transformed) or LDL-C (mg/dL) and baPWV in Model 1 without any covariate adjustment **(A)**, in Model 2 adjusting for covariates (age, sex, race, APOE-ɛ4 carrier status, cognitive status, education) **(B)**, and in Model 3 adjusting additionally for diabetes, hypertension, and mean arterial pressure **(C)**. The standardized odds ratio represents the difference in baPWV for a one-SD increment in each LDL cargo protein or a one-SD increment in LDL-C. P value (unadjusted) represents the statistical significance of the standardized beta coefficient. A p-value less than 0.05 suggests a one-SD increment in each LDL cargo protein (log_2_-transformed) or a one-SD increment in LDL-C (mg/dL) likely has a non-zero effect on baPWV.

Apolipoprotein B (apoB) in LDL demonstrated a significant association with baPWV in all models, including unadjusted Model 1 (Figure 1A; Supplementary Table 3A), partially adjusted Model 2 (Figure 1B; Supplementary Table 3B), and fully adjusted Model 3 (Figure 1C; Supplementary Table 3C). In Model 3, each SD increment of apoB in LDL was associated with an increase in baPWV of 103.0 cm/s (95% CI [43.3, 162.6]) (p-value of 0.001); each SD increment in LDL-C was associated with a smaller increase in baPWV of 54.3 cm/s (95% CI [-5.9, 114.6] (p-value of 0.08).

### Associations between LDL protein cargo or LDL-C and faPWV

**FIGURE 2:**
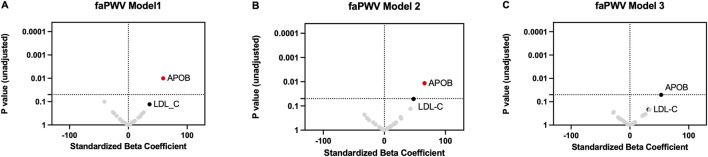
Association of LDL cargo proteins and LDL-C with faPWV. Linear regression was used to assess the associations between each LDL cargo protein (log_2_-transformed) or LDL-C (mg/dL) and faPWV in Model 1 without any covariate adjustment **(A)**, in Model 2 adjusting for covariates (age, sex, race, APOE-ɛ4 carrier status, cognitive status, education) **(B)**, and in Model 3 adjusting additionally for diabetes, hypertension, and mean arterial pressure **(C)**. The standardized odds ratio represents the difference in faPWV for a one-SD increment in each LDL cargo protein or a one-SD increment in LDL-C. P value (unadjusted) represents the statistical significance of the standardized beta coefficient. A p-value less than 0.05 suggests a one-SD increment in each LDL cargo protein (log_2_-transformed) or a one-SD increment in LDL-C (mg/dL) likely has a non-zero effect on faPWV.

ApoB in LDL demonstrated a significant association with faPWV in both unadjusted Model 1 (Figure 2A; Supplementary Table 4A) and partially adjusted Model 2 (Figure 2B; Supplementary Table 4B). However, this association was attenuated and became non-significant after full adjustment in Model 3 (Figure 2C; Supplementary Table 4C). In Model 3, each SD increment of apoB in LDL was associated with an increase in faPWV of 53.1 cm/s (95% CI [−0.1, 116.4]) (p-value of 0.05). Each SD increment in LDL-C was associated with a smaller increase in faPWV of 31.6 cm/s (95% CI [−19.2, 82.4] (p-value of 0.22).

### Associations between LDL protein cargo or LDL-C and cfPWV

**FIGURE 3:**
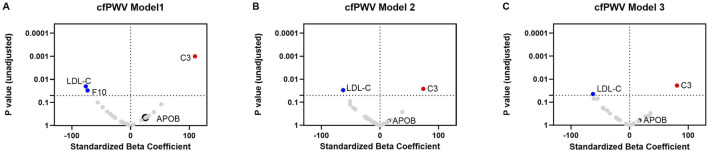
Association of LDL cargo proteins and LDL-C with cfPWV. Linear regression was used to assess the associations between each LDL cargo protein (log_2_-transformed) or LDL-C (mg/dL) and cfPWV in Model 1 without any covariate adjustment **(A)**, in Model 2 adjusting for covariates (age, sex, race, APOE-ɛ4 carrier status, cognitive status, education) **(B)**, and in Model 3 adjusting additionally for diabetes, hypertension, and mean arterial pressure **(C)**. The standardized odds ratio represents the difference in cfPWV for a one-SD increment in each LDL cargo protein or a one-SD increment in LDL-C. P value (unadjusted) represents the statistical significance of the standardized beta coefficient. A p-value less than 0.05 suggests a one-SD increment in each LDL cargo protein (log_2_-transformed) or a one-SD increment in LDL-C (mg/dL) likely has a non-zero effect on cfPWV.

C3 in LDL was significantly associated with cfPWV in unadjusted Model 1 (Figure 3A; Supplementary Table 5A) and partially adjusted Model 2 (Figure 3B; Supplementary Table 5B) and remained significant in the fully adjusted Model 3 (Figure 3C; Supplementary Table 5C), although the covariate adjustments in Models 2 and 3 attenuated the associations. In Model 3, each SD increment of C3 in LDL was associated with an increase in cfPWV of 80.9 cm/s (95% CI [14.0, 147.7]) (p-value of 0.02); each SD increment in LDL-C was associated with a decrease in cfPWV of 62.42 cm/s (95% CI [−123.0, −1.8] (p-value of 0.04).

## Discussion

In this exploratory study, we investigated LDL protein cargo and its associations with segment-specific PWV measures in older adults. We compared the effect sizes of these associations to those of LDL-C. We identified differential associations of LDL protein cargo and LDL-C with baPWV, faPWV, cfPWV, which measure arterial stiffness in peripheral and central arteries, peripheral artery, and central artery, respectively. ApoB in LDL and LDL-C each showed a positive association with baPWV, although the estimated effect size of apoB in LDL was greater than that of LDL-C. However, apoB in LDL was not associated with faPWV or cfPWV. Instead, we discovered that C3 in LDL was positively associated with cfPWV, and LDL-C was negatively associated cfPWV.

The observed positive associations between apoB in LDL or LDL-C with baPWV are consistent with previous studies (Zhao et al., 2014; Liu et al., 2022), confirming the strong association between baPWV and cardiovascular disease risk factors (Stone et al., 2023). Although baPWV showed a strong correlation with aortic PWV, and its variance was largely explained by aortic rather than peripheral PWV (Sugawara et al., 2005), an increasing consensus is that baPWV is a composite measure of central elastic arteries and muscular peripheral arteries in the lower extremities (Stone et al., 2023). Compared to large elastic central arteries, muscular peripheral arteries, which have smaller diameters and a higher proportion of stiffer and smooth muscle in their arterial walls, are more susceptible to lipid deposition and atherosclerosis (Cao et al., 2022; Reinilä et al., 1980). Although arteriosclerosis, an outside-in process characterized by degeneration of structural components in the vessel wall, is considered the main driver of arterial stiffness, atherosclerosis may also contribute by modifying the mechanical properties of the arterial wall, which may help explain the association between apoB in LDL or LDL-C with baPWV (Wilkinson et al., 2009). We also observed a stronger association of apoB in LDL with baPWV than that of LDL-C, likely because apoB in LDL is more reflective of the number of LDL particles than LDL-C.

We did not find a significant association of apoB in LDL with cfPWV, a measure of central (aortic) arterial stiffness. cfPWV is strongly associated with age and hypertension (Cecelja and Chowienczyk, 2009). We discovered that C3 in LDL was associated with cfPWV, independent of covariates, including age and hypertension. Baseline plasma C3 is strongly associated with baseline as well as the development of hypertension (Engström et al., 2007). A study of the mouse aorta suggested that a potential “outside-in” mechanism to explain the role of C3 in vascular stiffness, whereby C3 binds to elastic and collagen fibers in the adventitia, the outer layer of blood vessels, leading to vascular stiffness (Shields et al., 2011). Also, plasma C3 level was associated with cfPWV, although the association weakened and lost statistical significance after adjusting for more covariates (Jin et al., 2022). These observations support the role of C3 in the development of hypertension and central arterial stiffness (cfPWV).

The negative association observed between LDL-C and cfPWV is unexpected but aligns with two previous studies that reported similar non-significant inverse associations after adjusting for covariates (Heffernan et al., 2022; Liu et al., 2020). One attributed the negative association to the use of statins but noted an insignificant negative correlation between LDL-C and cfPWV in healthy subjects (Liu et al., 2020). These findings indicate that LDL-C may not have an overt detrimental role in central arterial stiffness (cfPWV) in older populations. It is unclear why there is a divergence in the association between apoB in LDL or LDL-C and cfPWV. Further analysis of LDL lipid cargo may offer insights.

This study has several strengths, including its innovative approach and methodologies for analyzing LDL cargo proteins, the physical isolation and measurement of LDL from a well-characterized cohort (the ARIC study), and the comparative analysis between baPWV and cfPWV, as well as between LDL cargo proteins and LDL-C, a widely recognized biomarker and therapeutic target for cardiovascular disease. Nonetheless, there are multiple limitations. First, due to the cross-sectional design, the study precludes the assessment of causal relationships between LDL cargo proteins and clinical outcomes. Second, the study is exploratory with a limited sample size. Replication of these findings in larger cohorts is necessary to ensure the generalizability of the results. Third, we utilized 10 μg of LDL proteins for mass spectrometry analysis; however, this approach did not account for individual variability in total LDL protein levels, as the same amount of protein was used across all samples. Further studies should address this variability to enhance the robustness of the findings. Fourth, it is possible that the identified LDL cargo proteins may also include non-LDL cargo proteins that coelute with the LDL fraction. To mitigate this, our analysis included only 28 proteins consistently identified in four previous studies referenced in the LDL proteome Watch. Fifth, LDL-C was calculated based on the Friedewald equation, instead of a direct measurement. There can be a significant level of disagreement between the LDL-C calculated by the Friedewald equation and a direct measurement, because the Friedewald equation does not account for Lp(a) cholesterol or the variability in cholesterol content of VLDL (Mora et al., 2009). However, the Friedewald equation remains the clinical gold standard for LDL-C calculation. Sixth, the study was conducted as part of a grant focused on neurocognitive outcomes; therefore, the study findings may not be generalizable to cardiovascular outcomes.

## Conclusion

We discovered differential associations of LDL cargo proteins with segment-specific PWVs. Our results implicate different pathophysiological mechanisms, atherosclerosis and the complement system, respectively, for arterial stiffness measured by baPWV and cfPWV.

## Data Availability

De-identified data are available from the ARIC coordinator center upon request made to the corresponding author.
