# Supplementary material for: Association of LDL protein cargo with segmental pulse wave velocity in older adults in the atherosclerosis risk in communities study
Source: Front Aging. 2026 May 22;7:1691150. doi: 10.3389/fragi.2026.1691150 (PMC13236892; doi:10.3389/fragi.2026.1691150)
Supplement: Supplementary file 1 [file Table1.docx]

**Supplementary Table 1**. Characteristics of eligible, included, and eligible but excluded study participants.

|  | Total Eligible | Included in the study | Eligible but not Included | P-value |
| --- | --- | --- | --- | --- |
| N | 340 | 65 | 275 |  |
| Age (year) | 75.9 (5.4) | 76.3 (5.4) | 75.8 (5.4) | 0.46 |
| BMI (kg/m^2) | 29.1 (5.4) | 29.7 (5.2) | 28.9 (5.4) | 0.34 |
| β-Amyloid Load on PET (SUVR) |  | 1.3 (0.3) |  | - |
| Gender |  |  |  |  |
| Female (n) | 193 (57%) | 35 (54%) | 158 (57%) | 0.7 |
| Male (n) | 147 (43%) | 30 (46%) | 117 (43%) |  |
| Race |  |  |  |  |
| Asian (n) | 2 (1%) | 0 (0%) | 2 (1%) | 0.11 |
| Black (n) | 141 (41%) | 20 (31%) | 121 (44%) |  |
| White (n) | 197 (58%) | 45 (69%) | 152 (55%) |  |
| Cognitive Status Diagnosis |  |  |  |  |
| Normal (n) | 249 (73%) | 47 (72%) | 202 (73%) | 0.97 |
| MCI (n) | 91 (27%) | 18 (28%) | 73 (27%) |  |
| APOE-ɛ4 Carrier Status |  |  |  |  |
| No (n) | 233 (69%) | 46 (71%) | 187 (68%) | 0.9 |
| Yes (n) | 102 (30%) | 18 (28%) | 84 (31%) |  |
| Missing (n) | 5 (1%) | 1 (2%) | 4 (1%) |  |
| Education Level |  |  |  |  |
| Basic-less than completed high school (n [%]) | 57 (17%) | 13 (20%) | 44 (16%) | 0.72 |
| Intermediate-completed high school or equivalent (n [%]) | 146 (43%) | 26 (40%) | 120 (44%) |  |
| High-completed at least some college (n [%]) | 137 (40%) | 26 (40%) | 111 (40%) |  |
| Pro-rated MMSE Score () | 27.2 (2.2) | 27.5 (2.1) | 27.1 (2.2) | 0.15 |
| Systolic Blood Pressure (mmHg) | 128.7 (16.1) | 126.8 (11.3) | 129.2 (17.0) | 0.27 |
| Diastolic Blood Pressure (mmHg) | 65.3 (10.5) | 64.8 (10.3) | 65.5 (10.5) | 0.66 |
| Total Intracranial Volume (mm^3) | 1372836.8 (155554.4) | 1387917.2 (160915.9) | 1369259.3 (154340.6) | 0.39 |
| Temporal-Parietal Meta-ROI Volume (mm^3) | 59048.1 (6805.0) | 60444.2 (7068.2) | 58718.1 (6712.1) | 0.07 |
| ARIC Field Center |  |  |  |  |
| Forsyth County, NC (n) | 73 (21%) | 19 (29%) | 54 (20%) | 0.1 |
| Jackson City, MS (n) | 135 (40%) | 19 (29%) | 116 (42%) |  |
| Washington County, MD (n) | 132 (39%) | 27 (42%) | 105 (38%) |  |
| Current Cigarette Smoking Status |  |  |  |  |
| Non-smoker (n) | 318 (94%) | 59 (91%) | 259 (94%) | 0.6 |
| Smoker (n) | 18 (5%) | 5 (8%) | 13 (5%) |  |
| Missing (n) | 4 (1%) | 1 (2%) | 3 (1%) |  |
| Diabetes Prevalence |  |  |  |  |
| No (n) | 213 (63%) | 43 (66%) | 170 (62%) | 0.75 |
| Yes (n) | 123 (36%) | 21 (32%) | 102 (37%) |  |
| Missing (n) | 4 (1%) | 1 (2%) | 3 (1%) |  |
| Hypertension Prevalence |  |  |  |  |
| No (n) | 94 (28%) | 20 (31%) | 74 (27%) | 0.59 |
| Yes (n) | 243 (71%) | 45 (69%) | 198 (72%) |  |
| Missing (n) | 3 (1%) | 0 (0%) | 3 (1%) |  |
| Coronary Heart Disease Prevalence |  |  |  |  |
| No (n) | 307 (90%) | 56 (86%) | 251 (91%) | 0.05 |
| Yes (n) | 26 (8%) | 9 (14%) | 17 (6%) |  |
| Missing (n) | 7 (2%) | 0 (0%) | 7 (3%) |  |
| Stroke Prevalence |  |  |  |  |
| No (n) | 327 (96%) | 63 (97%) | 264 (96%) | 0.87 |
| Yes (n) | 12 (4%) | 2 (3%) | 10 (4%) |  |
| Missing (n) | 1 (0%) | 0 (0%) | 1 (0%) |  |
| Definite or Possible Heart Failure Prevalence |  |  |  |  |
| No (n) | 299 (88%) | 58 (89%) | 241 (88%) | 0.89 |
| Yes (n) | 41 (12%) | 7 (11%) | 34 (12%) |  |
| Cholesterol Lowering Medication Use |  |  |  |  |
| No (n) | 156 (46%) | 31 (48%) | 125 (45%) | 0.85 |
| Yes (n) | 184 (54%) | 34 (52%) | 150 (55%) |  |
| Antihypertensive Medication Use |  |  |  |  |
| No (n) | 71 (21%) | 13 (20%) | 58 (21%) | 0.98 |
| Yes (n) | 269 (79%) | 52 (80%) | 217 (79%) |  |
| Statin Use |  |  |  |  |
| No (n) | 173 (51%) | 32 (49%) | 141 (51%) | 0.87 |
| Yes (n) | 167 (49%) | 33 (51%) | 134 (49%) |  |
| Carotid-femoral pulse wave velocity (cfPWV)(cm/s) | 1204.3(334.1) | 1093.4(264.4) | 1230.5(343.7) | 0.002 |
| Missing (n) | 25(7%) | 5(8%) | 20(7%) |  |
| Right brachial-ankle pulse wave velocity (RbaPWV)(cm/s) | 1728.3(334.6) | 1674.6(265.2) | 1740.8(348.0) | 0.19 |
| Missing (n) | 11(3%) | 3(5%) | 8(3%) |  |
| Femoral-right ankle pulse wave velocity (RfaPWV)(cm/s) | 1609.0(188.9) | 1075.9(188.2) | 1067.4(189.4) | 0.86 |
| Missing (n) | 25(7%) | 5(8%) | 20(7%) |  |
| Mean Arterial Pressure(mmHg) | 86.5(10.8) | 85.4(9.3) | 86.7(11.1) | 0.66 |
| Missing (n) | 2(0.6%) | 0(0%) | 2(0.7%) |  |

**Supplementary Table 2A.** Relative abundance of the 28 proteins (Ranked by mean log2 intensity).

| Proteins | Mean of Log2 Intensity |
| --- | --- |
| APOC4 | 12.34974988 |
| IGKC | 12.35671822 |
| PPBP | 12.44762471 |
| CAMP | 13.15968602 |
| APOH | 13.87013426 |
| APOC1 | 13.89321167 |
| APOF | 14.59569092 |
| F10 | 14.60360898 |
| APOC3 | 15.31492702 |
| APOA2 | 15.60937519 |
| HPR | 15.82925274 |
| APOM | 16.33570043 |
| APOL1 | 16.39518569 |
| C3 | 16.9143214 |
| APOA1 | 17.08394567 |
| HP | 17.23729635 |
| CLU | 17.45779013 |
| APOD | 17.58360105 |
| IGHA1 | 17.61552747 |
| PON1 | 17.65552911 |
| F2 | 17.80216299 |
| IGHA2 | 18.23127125 |
| ALB | 18.29761114 |
| APOE | 18.51716918 |
| FGG | 18.70211608 |
| APOB | 18.75716333 |
| VTN | 19.88327539 |
| FGA | 21.43953663 |

**Supplementary Table 2B.** Pearson correlation between LDL protein cargo and LDL-C.

| Proteins | Correlation | P-value |
| --- | --- | --- |
| APOE | 0.4084 | 0.0006 |
| APOC4 | 0.4055 | 0.001 |
| APOM | 0.3798 | 0.0016 |
| APOL1 | 0.3655 | 0.0026 |
| CAMP | 0.3431 | 0.0054 |
| HPR | 0.2816 | 0.0227 |
| APOB | 0.2556 | 0.0395 |
| PON1 | 0.203 | 0.1051 |
| APOA2 | 0.1628 | 0.196 |
| APOC1 | 0.1296 | 0.3047 |
| HP | 0.1264 | 0.3172 |
| APOC3 | 0.1217 | 0.3355 |
| APOD | 0.1182 | 0.3497 |
| VTN | 0.0919 | 0.4678 |
| ALB | 0.0628 | 0.6204 |
| F10 | 0.0074 | 0.9535 |
| IGHA1 | 0.0043 | 0.9729 |
| F2 | -0.0025 | 0.9844 |
| IGKC | -0.0233 | 0.8547 |
| FGA | -0.029 | 0.8195 |
| APOF | -0.0338 | 0.79 |
| CLU | -0.0642 | 0.6125 |
| FGG | -0.0804 | 0.5256 |
| IGHA2 | -0.0843 | 0.5057 |
| C3 | -0.0896 | 0.4794 |
| APOH | -0.1124 | 0.3739 |
| PPBP | -0.1533 | 0.2535 |
| APOA1 | -0.2238 | 0.073 |

**Supplementary Table 3A.** Association of 28 LDL cargo proteins and baPWV(Model 1).

| protein or LDL-C | coefficient | 95% CI of coefficient | pvalue | BH_pvalue | standardized_coefficient | standardized 95% CI of coefficient |
| --- | --- | --- | --- | --- | --- | --- |
| APOB | 182.4 | 65.1,299.7 | 0.003 | 0.08 | 96.6 | 34.5,158.7 |
| ALB | 162.2 | -37.4,361.7 | 0.11 | 0.67 | 53.7 | -12.4,119.9 |
| VTN | -114.3 | -258.6,29.9 | 0.12 | 0.67 | -52.3 | -118.3,13.7 |
| PON1 | 90.2 | -25.9,206.2 | 0.13 | 0.67 | 51.3 | -14.7,117.3 |
| APOC3 | -42.9 | -99,13.1 | 0.13 | 0.67 | -50.9 | -117.4,15.5 |
| APOE | 87.5 | -29.4,204.5 | 0.14 | 0.67 | 49.3 | -16.5,115.1 |
| C3 | 113 | -71.3,297.3 | 0.22 | 0.8 | 40.4 | -25.5,106.2 |
| LDL_C | 1.1 | -0.8,2.9 | 0.25 | 0.8 | 38.8 | -27.4,105.1 |
| F2 | -54.6 | -149.8,40.6 | 0.26 | 0.8 | -39.3 | -107.7,29.2 |
| APOC1 | -41.8 | -118.2,34.5 | 0.28 | 0.8 | -39 | -110,32.1 |
| IGHA2 | -62 | -181.7,57.8 | 0.3 | 0.8 | -34.8 | -102,32.4 |
| PPBP | -35.4 | -112.6,41.7 | 0.36 | 0.84 | -34.1 | -108.4,40.2 |
| F10 | -75.6 | -256.4,105.3 | 0.41 | 0.84 | -28 | -94.9,39 |
| APOA1 | 76.5 | -107.2,260.3 | 0.41 | 0.84 | 28.9 | -40.5,98.3 |
| APOF | -36.3 | -137.6,64.9 | 0.48 | 0.88 | -24.3 | -91.9,43.4 |
| APOA2 | 85.9 | -164.5,336.3 | 0.49 | 0.88 | 23.6 | -45.2,92.4 |
| APOC4 | -18.2 | -80.4,43.9 | 0.56 | 0.88 | -21.1 | -93.1,50.9 |
| CLU | 62.5 | -158.4,283.4 | 0.57 | 0.88 | 20.3 | -51.5,92.1 |
| APOH | -28 | -128.4,72.4 | 0.58 | 0.88 | -19 | -87.2,49.2 |
| HPR | 15.2 | -64,94.4 | 0.7 | 0.94 | 12.8 | -54.1,79.8 |
| APOL1 | -20.3 | -154.3,113.7 | 0.76 | 0.94 | -10.1 | -76.8,56.6 |
| IGKC | -12.6 | -96,70.8 | 0.76 | 0.94 | -10.2 | -77.9,57.4 |
| CAMP | 10.4 | -59.8,80.5 | 0.77 | 0.94 | 10.3 | -59.3,79.8 |
| APOM | 20.3 | -123.7,164.2 | 0.78 | 0.94 | 9.5 | -58.2,77.3 |
| FGG | -10.7 | -136.1,114.7 | 0.87 | 0.98 | -5.8 | -74.5,62.8 |
| IGHA1 | -7.6 | -116.8,101.7 | 0.89 | 0.98 | -4.7 | -72.4,63 |
| FGA | -1.7 | -116.2,112.8 | 0.98 | 0.98 | -1 | -68.9,66.8 |
| HP | 1.2 | -113.1,115.6 | 0.98 | 0.98 | 0.7 | -67.7,69.2 |
| APOD | -1.5 | -155,152 | 0.98 | 0.98 | -0.7 | -71.9,70.5 |

**Supplementary Table 3B.** Association of 28 LDL cargo proteins and baPWV (Model 2).

| protein or LDL-C | coefficient | 95% CI of coefficient | pvalue | BH_pvalue | standardized_coefficient | standardized 95% CI of coefficient |
| --- | --- | --- | --- | --- | --- | --- |
| APOB | 216.5 | 110.6,322.3 | p<0.001 | 0.004 | 114.6 | 58.6,170.7 |
| LDL_C | 1.9 | 0.3,3.5 | 0.02 | 0.32 | 67.3 | 10.2,124.5 |
| APOE | 94.8 | -22,211.6 | 0.11 | 0.71 | 53.4 | -12.4,119.1 |
| PPBP | -57 | -127.6,13.6 | 0.11 | 0.71 | -54.8 | -122.8,13.1 |
| PON1 | 85.9 | -23.8,195.6 | 0.12 | 0.71 | 48.9 | -13.5,111.3 |
| IGHA2 | -54.7 | -164.7,55.3 | 0.32 | 0.82 | -30.7 | -92.5,31.1 |
| APOF | -45.2 | -146.5,56.2 | 0.37 | 0.82 | -30.2 | -97.8,37.5 |
| APOD | -63.5 | -209,81.9 | 0.38 | 0.82 | -29.5 | -96.9,38 |
| F10 | 74.3 | -98.5,247.2 | 0.39 | 0.82 | 27.5 | -36.5,91.5 |
| HPR | 33.1 | -46.4,112.7 | 0.41 | 0.82 | 28 | -39.2,95.3 |
| APOM | 54.6 | -78.6,187.8 | 0.41 | 0.82 | 25.7 | -37,88.4 |
| FGA | -43 | -148.9,62.9 | 0.42 | 0.82 | -25.5 | -88.2,37.3 |
| F2 | -31.3 | -117.3,54.7 | 0.47 | 0.82 | -22.5 | -84.3,39.3 |
| APOA1 | 59.8 | -118.7,238.3 | 0.5 | 0.82 | 22.6 | -44.8,90 |
| ALB | 66.1 | -131.9,264.1 | 0.51 | 0.82 | 21.9 | -43.7,87.5 |
| APOC3 | -18.2 | -73.4,37 | 0.51 | 0.82 | -21.6 | -87.1,43.9 |
| HP | -36 | -147.3,75.2 | 0.52 | 0.82 | -21.6 | -88.2,45 |
| APOH | -26.7 | -117.9,64.5 | 0.56 | 0.82 | -18.1 | -80.1,43.8 |
| APOC1 | -20.5 | -90.8,49.9 | 0.56 | 0.82 | -19 | -84.6,46.5 |
| CAMP | 19 | -46.7,84.6 | 0.56 | 0.82 | 18.8 | -46.3,83.9 |
| APOL1 | 34.6 | -94.8,163.9 | 0.59 | 0.82 | 17.2 | -47.1,81.5 |
| APOA2 | 52.7 | -173.3,278.7 | 0.64 | 0.85 | 14.5 | -47.6,76.6 |
| C3 | 36.2 | -144.4,216.7 | 0.69 | 0.85 | 12.9 | -51.6,77.4 |
| FGG | -21.6 | -136.7,93.4 | 0.71 | 0.85 | -11.8 | -74.8,51.1 |
| VTN | -24.6 | -173.8,124.7 | 0.74 | 0.86 | -11.2 | -79.5,57.1 |
| IGHA1 | 7.9 | -92,107.7 | 0.87 | 0.97 | 4.9 | -57,66.8 |
| CLU | -10.6 | -220,198.7 | 0.92 | 0.97 | -3.5 | -71.5,64.6 |
| APOC4 | 2 | -53.1,57 | 0.94 | 0.97 | 2.3 | -61.4,66 |
| IGKC | 1.2 | -74.3,76.8 | 0.97 | 0.97 | 1 | -60.3,62.3 |

**Supplementary Table 3C.** Association of 28 LDL cargo proteins and baPWV (Model 3).

| protein or LDL-C | coefficient | 95% CI of coefficient | pvalue | BH_pvalue | standardized_coefficient | standardized 95% CI of coefficient |
| --- | --- | --- | --- | --- | --- | --- |
| APOB | 194.5 | 81.8,307.2 | 0.001 | 0.03 | 103 | 43.3,162.6 |
| LDL_C | 1.5 | -0.2,3.2 | 0.08 | 0.74 | 54.3 | -5.9,114.6 |
| APOD | -112.3 | -252.5,28 | 0.11 | 0.74 | -52.1 | -117.1,13 |
| PPBP | -55.2 | -124.5,14 | 0.12 | 0.74 | -53.2 | -119.8,13.5 |
| IGHA2 | -77 | -183.7,29.8 | 0.15 | 0.74 | -43.2 | -103.1,16.7 |
| APOE | 81.3 | -32.3,194.9 | 0.16 | 0.74 | 45.7 | -18.2,109.7 |
| PON1 | 70.3 | -37.2,177.8 | 0.19 | 0.74 | 40 | -21.1,101.2 |
| FGA | -61.6 | -164.6,41.3 | 0.23 | 0.74 | -36.5 | -97.5,24.5 |
| APOH | -52.5 | -142.8,37.9 | 0.25 | 0.74 | -35.6 | -97,25.7 |
| VTN | -84.9 | -234.8,64.9 | 0.26 | 0.74 | -38.9 | -107.5,29.7 |
| HPR | 44.3 | -37.4,126 | 0.28 | 0.74 | 37.5 | -31.6,106.5 |
| APOA1 | 82.5 | -94,259 | 0.35 | 0.84 | 31.2 | -35.5,97.8 |
| F10 | 73.9 | -101.8,249.6 | 0.4 | 0.84 | 27.4 | -37.7,92.4 |
| CLU | -84.7 | -294.4,124.9 | 0.42 | 0.84 | -27.5 | -95.7,40.6 |
| APOL1 | 49 | -78.1,176 | 0.44 | 0.84 | 24.3 | -38.8,87.5 |
| APOA2 | 78.1 | -153.3,309.5 | 0.5 | 0.84 | 21.5 | -42.1,85.1 |
| FGG | -37 | -150.1,76.2 | 0.51 | 0.84 | -20.2 | -82.2,41.7 |
| APOF | -32.3 | -132.2,67.7 | 0.52 | 0.84 | -21.5 | -88.3,45.2 |
| F2 | -24.5 | -109.7,60.8 | 0.57 | 0.86 | -17.6 | -78.8,43.7 |
| APOM | 25.1 | -105.6,155.9 | 0.7 | 0.96 | 11.8 | -49.7,73.4 |
| HP | -21 | -131.6,89.6 | 0.7 | 0.96 | -12.6 | -78.8,53.7 |
| C3 | 31.9 | -151.9,215.7 | 0.73 | 0.96 | 11.4 | -54.3,77.1 |
| APOC1 | -9.9 | -79.8,60 | 0.78 | 0.97 | -9.3 | -74.3,55.8 |
| IGHA1 | -10 | -107.7,87.7 | 0.84 | 0.97 | -6.2 | -66.8,54.3 |
| APOC3 | -4.7 | -59.2,49.8 | 0.86 | 0.97 | -5.6 | -70.3,59.1 |
| CAMP | 4.6 | -61.4,70.7 | 0.89 | 0.97 | 4.6 | -60.8,70.1 |
| ALB | 10 | -186.8,206.8 | 0.92 | 0.97 | 3.3 | -61.9,68.5 |
| APOC4 | -2 | -54.5,50.4 | 0.94 | 0.97 | -2.4 | -63.1,58.3 |
| IGKC | -0.3 | -73.4,72.8 | p>0.99 | p>0.99 | -0.2 | -59.5,59 |

**Supplementary Table 4A.** Association of 28 LDL cargo proteins and faPWV (Model 1).

| protein or LDL-C | coefficient | 95% CI of coefficient | pvalue | BH_pvalue | standardized_coefficient | standardized 95% CI of coefficient |
| --- | --- | --- | --- | --- | --- | --- |
| APOB | 111.7 | 26.4,197.1 | 0.01 | 0.32 | 59.2 | 14,104.3 |
| APOC3 | -34.2 | -75.5,7.1 | 0.1 | 0.99 | -40.6 | -89.5,8.4 |
| LDL_C | 1 | -0.3,2.3 | 0.13 | 0.99 | 36 | -11.1,83 |
| HP | -43.8 | -123.9,36.3 | 0.28 | 0.99 | -26.2 | -74.1,21.7 |
| APOH | 38.2 | -32.8,109.2 | 0.29 | 0.99 | 25.9 | -22.3,74.2 |
| IGHA2 | -43.1 | -128.4,42.1 | 0.32 | 0.99 | -24.2 | -72.1,23.7 |
| APOE | 40.7 | -44.6,126 | 0.34 | 0.99 | 22.9 | -25.1,70.9 |
| APOF | -29.9 | -102.7,42.9 | 0.41 | 0.99 | -20 | -68.6,28.7 |
| CAMP | 20.5 | -30.5,71.6 | 0.42 | 0.99 | 20.4 | -30.3,71 |
| ALB | 51.5 | -93.4,196.4 | 0.48 | 0.99 | 17.1 | -31,65.1 |
| IGKC | -17.7 | -78.8,43.4 | 0.56 | 0.99 | -14.3 | -63.9,35.2 |
| F2 | -19.7 | -90.4,50.9 | 0.58 | 0.99 | -14.2 | -64.9,36.6 |
| FGG | 20 | -70.7,110.8 | 0.66 | 0.99 | 11 | -38.7,60.7 |
| APOD | 24 | -85.9,133.8 | 0.66 | 0.99 | 11.1 | -39.8,62 |
| APOC1 | 11.5 | -43.3,66.3 | 0.68 | 0.99 | 10.7 | -40.3,61.7 |
| APOC4 | -8.8 | -53.4,35.8 | 0.69 | 0.99 | -10.2 | -61.8,41.4 |
| PPBP | -11.1 | -67.7,45.6 | 0.7 | 0.99 | -10.6 | -65.2,43.9 |
| FGA | 15.5 | -66.9,97.9 | 0.71 | 0.99 | 9.2 | -39.7,58 |
| HPR | 9.7 | -48.4,67.9 | 0.74 | 0.99 | 8.2 | -40.9,57.4 |
| APOM | 14.4 | -90.7,119.6 | 0.78 | 0.99 | 6.8 | -42.7,56.3 |
| VTN | 13.2 | -91.4,117.7 | 0.8 | 0.99 | 6 | -41.8,53.9 |
| APOA2 | -22.8 | -206.9,161.2 | 0.8 | 0.99 | -6.3 | -56.9,44.3 |
| IGHA1 | 5.7 | -73,84.5 | 0.88 | 0.99 | 3.5 | -45.3,52.4 |
| C3 | 8.7 | -126.9,144.3 | 0.9 | 0.99 | 3.1 | -45.3,51.5 |
| F10 | -5.7 | -137.7,126.4 | 0.93 | 0.99 | -2.1 | -51,46.8 |
| APOA1 | -4.2 | -140.9,132.5 | 0.95 | 0.99 | -1.6 | -53.2,50.1 |
| APOL1 | -2 | -98.5,94.4 | 0.97 | 0.99 | -1 | -49,46.9 |
| PON1 | 1.2 | -83.9,86.2 | 0.98 | 0.99 | 0.7 | -47.7,49 |
| CLU | 1.4 | -155.6,158.3 | 0.99 | 0.99 | 0.4 | -50.6,51.4 |

**Supplementary Table 4B.** Association of 28 LDL cargo proteins and faPWV (Model 2).

| protein or LDL-C | coefficient | 95% CI of coefficient | pvalue | BH_pvalue | standardized_coefficient | standardized 95% CI of coefficient |
| --- | --- | --- | --- | --- | --- | --- |
| APOB | 124.2 | 29,219.3 | 0.01 | 0.34 | 65.7 | 15.3,116.1 |
| LDL_C | 1.3 | 0,2.7 | 0.05 | 0.74 | 47.9 | -0.3,96 |
| APOE | 76 | -23.5,175.5 | 0.13 | 0.98 | 42.8 | -13.2,98.8 |
| HP | -54.3 | -144,35.4 | 0.23 | 0.98 | -32.5 | -86.2,21.2 |
| APOH | 39.5 | -34.4,113.4 | 0.29 | 0.98 | 26.8 | -23.4,77 |
| APOC3 | -23.9 | -71.4,23.6 | 0.32 | 0.98 | -28.4 | -84.7,28 |
| VTN | 60 | -62.4,182.5 | 0.33 | 0.98 | 27.5 | -28.6,83.5 |
| APOF | -37.5 | -122.8,47.8 | 0.38 | 0.98 | -25.1 | -82,31.9 |
| IGHA2 | -32.6 | -123.8,58.7 | 0.48 | 0.98 | -18.3 | -69.5,32.9 |
| F10 | 51.8 | -95.3,198.9 | 0.48 | 0.98 | 19.2 | -35.3,73.6 |
| CAMP | 16.9 | -38.7,72.5 | 0.54 | 0.98 | 16.8 | -38.3,71.8 |
| APOC1 | 16.2 | -41.3,73.6 | 0.57 | 0.98 | 15 | -38.4,68.5 |
| IGKC | -17.7 | -81.4,46.1 | 0.58 | 0.98 | -14.3 | -66.1,37.4 |
| FGG | 19.9 | -77.4,117.2 | 0.68 | 0.98 | 10.9 | -42.4,64.1 |
| HPR | 12.7 | -55.8,81.1 | 0.71 | 0.98 | 10.7 | -47.2,68.6 |
| APOA2 | -30.9 | -221.5,159.6 | 0.75 | 0.98 | -8.5 | -60.9,43.9 |
| PPBP | -8.4 | -72.2,55.4 | 0.79 | 0.98 | -8.1 | -69.4,53.3 |
| IGHA1 | 11 | -72.7,94.8 | 0.79 | 0.98 | 6.8 | -45.1,58.8 |
| FGA | 11.2 | -78.1,100.5 | 0.8 | 0.98 | 6.6 | -46.3,59.6 |
| F2 | -8.8 | -82,64.4 | 0.81 | 0.98 | -6.3 | -58.9,46.3 |
| ALB | 17.4 | -147.5,182.3 | 0.83 | 0.98 | 5.8 | -48.9,60.4 |
| APOM | 11.1 | -103,125.1 | 0.85 | 0.98 | 5.2 | -48.5,58.9 |
| C3 | 14.4 | -138.8,167.7 | 0.85 | 0.98 | 5.1 | -49.6,59.9 |
| APOA1 | 10.6 | -142.7,163.8 | 0.89 | 0.98 | 4 | -53.9,61.9 |
| CLU | -10.9 | -178.8,157 | 0.9 | 0.98 | -3.6 | -58.1,51 |
| PON1 | -3.5 | -98.6,91.5 | 0.94 | 0.98 | -2 | -56.1,52 |
| APOC4 | -1.7 | -48.1,44.7 | 0.94 | 0.98 | -2 | -55.7,51.7 |
| APOL1 | -2.5 | -111,106.1 | 0.96 | 0.98 | -1.2 | -55.2,52.8 |
| APOD | 1.8 | -117.5,121.1 | 0.98 | 0.98 | 0.8 | -54.5,56.2 |

**Supplementary Table 4C.** Association of 28 LDL cargo proteins and faPWV (Model 3).

| protein or LDL-C | coefficient | 95% CI of coefficient | pvalue | BH_pvalue | standardized_coefficient | standardized 95% CI of coefficient |
| --- | --- | --- | --- | --- | --- | --- |
| APOB | 100.4 | -0.3,201 | 0.05 | 0.97 | 53.1 | -0.1,106.4 |
| LDL_C | 0.9 | -0.5,2.3 | 0.22 | 0.97 | 31.6 | -19.2,82.4 |
| APOE | 59.4 | -37.3,156 | 0.22 | 0.97 | 33.4 | -21,87.8 |
| IGHA2 | -49 | -137.7,39.7 | 0.27 | 0.97 | -27.5 | -77.3,22.3 |
| HP | -47.1 | -136.1,42 | 0.29 | 0.97 | -28.2 | -81.5,25.1 |
| F10 | 71.5 | -77.4,220.4 | 0.34 | 0.97 | 26.5 | -28.6,81.6 |
| APOC1 | 25.4 | -31,81.9 | 0.37 | 0.97 | 23.7 | -28.9,76.2 |
| APOH | 31 | -43,105.1 | 0.4 | 0.97 | 21.1 | -29.2,71.4 |
| CLU | -61.9 | -230.2,106.4 | 0.46 | 0.97 | -20.1 | -74.8,34.6 |
| APOD | -39.1 | -156.1,78 | 0.5 | 0.97 | -18.1 | -72.4,36.2 |
| APOF | -25.2 | -109.1,58.7 | 0.55 | 0.97 | -16.8 | -72.9,39.2 |
| APOC3 | -13.9 | -61,33.2 | 0.56 | 0.97 | -16.5 | -72.4,39.4 |
| IGKC | -16.8 | -78.9,45.3 | 0.59 | 0.97 | -13.6 | -64,36.7 |
| PON1 | -18.1 | -110,73.7 | 0.69 | 0.97 | -10.3 | -62.6,41.9 |
| APOA2 | -31.8 | -226.3,162.7 | 0.74 | 0.97 | -8.7 | -62.2,44.7 |
| CAMP | 8.9 | -46.1,63.9 | 0.74 | 0.97 | 8.9 | -45.7,63.4 |
| ALB | -25.9 | -188.7,137 | 0.75 | 0.97 | -8.6 | -62.6,45.4 |
| APOM | -14.9 | -126.8,97 | 0.79 | 0.97 | -7 | -59.7,45.6 |
| VTN | 16.5 | -108.9,142 | 0.79 | 0.97 | 7.6 | -49.9,65 |
| C3 | 18.4 | -137.9,174.6 | 0.81 | 0.97 | 6.6 | -49.3,62.4 |
| HPR | 8.1 | -61.9,78.2 | 0.82 | 0.97 | 6.9 | -52.4,66.1 |
| FGA | -9.2 | -96.5,78.1 | 0.83 | 0.97 | -5.5 | -57.2,46.3 |
| PPBP | -4.7 | -68.8,59.3 | 0.88 | 0.97 | -4.6 | -66.2,57.1 |
| APOA1 | 9.6 | -141.7,161 | 0.9 | 0.97 | 3.6 | -53.5,60.8 |
| APOC4 | -2.6 | -47.6,42.3 | 0.91 | 0.97 | -3.1 | -55.1,49 |
| APOL1 | -3.6 | -110.1,102.9 | 0.95 | 0.97 | -1.8 | -54.8,51.2 |
| IGHA1 | -2.1 | -84.1,79.8 | 0.96 | 0.97 | -1.3 | -52.1,49.5 |
| FGG | -2.3 | -98.7,94.1 | 0.96 | 0.97 | -1.2 | -54,51.5 |
| F2 | -1.2 | -73.6,71.2 | 0.97 | 0.97 | -0.8 | -52.9,51.2 |

**Supplementary Table 5A.** Association of 28 LDL cargo proteins and cfPWV (Model 1).

| protein or LDL-C | coefficient | 95% CI of coefficient | pvalue | BH_pvalue | standardized_coefficient | standardized 95% CI of coefficient |
| --- | --- | --- | --- | --- | --- | --- |
| C3 | 307.1 | 127.2,486.9 | 0.001 | 0.03 | 109.7 | 45.5,173.9 |
| LDL_C | -2.1 | -3.9,-0.3 | 0.02 | 0.29 | -76.3 | -140.3,-12.4 |
| F10 | -198.1 | -377.8,-18.4 | 0.03 | 0.3 | -73.3 | -139.8,-6.8 |
| APOC3 | -47.2 | -103.8,9.4 | 0.1 | 0.59 | -56 | -123.2,11.1 |
| ALB | 158 | -40.7,356.7 | 0.12 | 0.59 | 52.4 | -13.5,118.2 |
| IGHA1 | -76.9 | -183.9,30 | 0.16 | 0.59 | -47.7 | -114,18.6 |
| VTN | -103.4 | -247.6,40.9 | 0.16 | 0.59 | -47.3 | -113.3,18.7 |
| APOL1 | -96.1 | -231.9,39.8 | 0.16 | 0.59 | -47.8 | -115.4,19.8 |
| CAMP | -40.1 | -109,28.8 | 0.25 | 0.67 | -39.8 | -108.1,28.5 |
| APOA1 | 105 | -79.2,289.2 | 0.26 | 0.67 | 39.7 | -29.9,109.3 |
| APOH | -50.8 | -150.9,49.3 | 0.31 | 0.67 | -34.5 | -102.5,33.5 |
| F2 | -45.3 | -141.2,50.5 | 0.35 | 0.67 | -32.6 | -101.5,36.3 |
| FGA | 52.3 | -62.4,167 | 0.37 | 0.67 | 31 | -37,99 |
| CLU | 94.3 | -122.2,310.9 | 0.39 | 0.67 | 30.7 | -39.7,101 |
| APOM | -61.5 | -208.7,85.7 | 0.41 | 0.67 | -28.9 | -98.2,40.3 |
| APOA2 | 99.6 | -155.3,354.5 | 0.44 | 0.67 | 27.4 | -42.7,97.4 |
| APOF | 39.2 | -62.7,141.2 | 0.44 | 0.67 | 26.2 | -41.9,94.3 |
| APOB | 47.5 | -77.6,172.7 | 0.45 | 0.67 | 25.2 | -41.1,91.4 |
| IGKC | -32.6 | -120.6,55.4 | 0.46 | 0.67 | -26.4 | -97.8,44.9 |
| HP | 41.9 | -71.2,155.1 | 0.46 | 0.67 | 25.1 | -42.6,92.8 |
| PON1 | 32.1 | -85,149.3 | 0.59 | 0.81 | 18.3 | -48.4,84.9 |
| APOC4 | -14 | -80.3,52.3 | 0.67 | 0.89 | -16.2 | -92.9,60.6 |
| APOC1 | -13.2 | -91.8,65.4 | 0.74 | 0.93 | -12.3 | -85.5,60.9 |
| IGHA2 | -12.6 | -133,107.7 | 0.83 | 0.94 | -7.1 | -74.7,60.5 |
| HPR | -5.5 | -90.2,79.2 | 0.9 | 0.94 | -4.7 | -76.3,66.9 |
| PPBP | 4.8 | -71,80.6 | 0.9 | 0.94 | 4.6 | -68.4,77.5 |
| FGG | 7.7 | -118.7,134.1 | 0.9 | 0.94 | 4.2 | -65,73.4 |
| APOD | -8 | -160.9,144.9 | 0.92 | 0.94 | -3.7 | -74.6,67.2 |
| APOE | -4.7 | -123.9,114.6 | 0.94 | 0.94 | -2.6 | -69.8,64.5 |

**Supplementary Table 5B.** Association of 28 LDL cargo proteins and cfPWV (Model 2).

| protein or LDL-C | coefficient | 95% CI of coefficient | pvalue | BH_pvalue | standardized_coefficient | standardized 95% CI of coefficient |
| --- | --- | --- | --- | --- | --- | --- |
| C3 | 206.7 | 26,387.5 | 0.03 | 0.43 | 73.9 | 9.3,138.4 |
| LDL_C | -1.7 | -3.3,-0.2 | 0.03 | 0.43 | -62.9 | -119.2,-6.5 |
| IGHA1 | -82.3 | -177.6,13 | 0.09 | 0.86 | -51 | -110.1,8 |
| APOE | -90.7 | -208,26.6 | 0.13 | 0.92 | -51.1 | -117.1,15 |
| APOC3 | -38.5 | -94.3,17.3 | 0.17 | 0.93 | -45.7 | -111.9,20.5 |
| F10 | -113.7 | -288.8,61.5 | 0.2 | 0.93 | -42.1 | -106.9,22.8 |
| APOF | 56.7 | -43.9,157.3 | 0.26 | 0.93 | 37.9 | -29.3,105.1 |
| CAMP | -25.9 | -90.2,38.5 | 0.42 | 0.93 | -25.6 | -89.4,38.2 |
| APOH | -34.8 | -124.8,55.1 | 0.44 | 0.93 | -23.7 | -84.8,37.4 |
| F2 | -31.3 | -117.4,54.7 | 0.47 | 0.93 | -22.5 | -84.4,39.3 |
| APOD | -50.9 | -193.2,91.5 | 0.48 | 0.93 | -23.6 | -89.6,42.4 |
| APOM | -42.9 | -178,92.2 | 0.53 | 0.93 | -20.2 | -83.8,43.4 |
| IGHA2 | -33.8 | -142,74.4 | 0.53 | 0.93 | -19 | -79.7,41.7 |
| ALB | 55.6 | -137.6,248.8 | 0.57 | 0.93 | 18.4 | -45.6,82.5 |
| FGG | -27.3 | -143.2,88.6 | 0.64 | 0.93 | -14.9 | -78.4,48.5 |
| APOB | 27.6 | -90.6,145.7 | 0.64 | 0.93 | 14.6 | -47.9,77.1 |
| APOA2 | 47.9 | -178.2,274 | 0.67 | 0.93 | 13.2 | -49,75.3 |
| APOC1 | 14.7 | -56.9,86.2 | 0.68 | 0.93 | 13.6 | -53,80.3 |
| APOA1 | 32.9 | -146.2,211.9 | 0.71 | 0.93 | 12.4 | -55.2,80.1 |
| IGKC | -13.2 | -90.8,64.4 | 0.73 | 0.93 | -10.7 | -73.6,52.2 |
| HPR | 14.2 | -70.9,99.3 | 0.74 | 0.93 | 12 | -59.9,84 |
| APOL1 | -16.4 | -147.3,114.5 | 0.8 | 0.93 | -8.2 | -73.3,56.9 |
| VTN | -16.7 | -163.5,130.2 | 0.82 | 0.93 | -7.6 | -74.8,59.6 |
| HP | -10.6 | -119.6,98.3 | 0.85 | 0.93 | -6.4 | -71.6,58.9 |
| PON1 | 10.5 | -99.6,120.7 | 0.85 | 0.93 | 6 | -56.7,68.7 |
| APOC4 | 5.7 | -54.4,65.8 | 0.85 | 0.93 | 6.6 | -63,76.2 |
| FGA | -7.7 | -114.4,99 | 0.89 | 0.93 | -4.6 | -67.8,58.7 |
| PPBP | -4.8 | -77.6,68.1 | 0.9 | 0.93 | -4.6 | -74.6,65.5 |
| CLU | 3.4 | -197.9,204.7 | 0.97 | 0.97 | 1.1 | -64.3,66.5 |

**Supplementary Table 5C.** Association of 28 LDL cargo proteins and cfPWV (Model 3).

| protein or LDL-C | coefficient | 95% CI of coefficient | pvalue | BH_pvalue | standardized_coefficient | standardized 95% CI of coefficient |
| --- | --- | --- | --- | --- | --- | --- |
| C3 | 226.3 | 39.1,413.6 | 0.02 | 0.51 | 80.9 | 14,147.7 |
| LDL_C | -1.7 | -3.4,0 | 0.04 | 0.51 | -62.4 | -123.1,-1.8 |
| IGHA1 | -87.9 | -183,7.2 | 0.07 | 0.51 | -54.5 | -113.4,4.5 |
| F10 | -164.1 | -342.4,14.1 | 0.07 | 0.51 | -60.8 | -126.8,5.2 |
| APOE | -80.9 | -198.5,36.7 | 0.17 | 0.93 | -45.5 | -111.7,20.7 |
| APOC3 | -37.1 | -93.5,19.4 | 0.19 | 0.93 | -44 | -111,23 |
| APOH | -56.3 | -149.5,36.9 | 0.23 | 0.93 | -38.2 | -101.6,25.1 |
| APOF | 53.3 | -48.5,155.1 | 0.3 | 0.93 | 35.6 | -32.4,103.6 |
| CAMP | -33.8 | -99.5,31.9 | 0.31 | 0.93 | -33.5 | -98.6,31.7 |
| HPR | 38 | -49.6,125.6 | 0.39 | 0.93 | 32.1 | -41.9,106.2 |
| APOA2 | 97.3 | -138,332.6 | 0.41 | 0.93 | 26.7 | -37.9,91.4 |
| APOA1 | 73.4 | -108.2,255.1 | 0.42 | 0.93 | 27.7 | -40.9,96.4 |
| IGHA2 | -40.7 | -149.6,68.2 | 0.46 | 0.93 | -22.8 | -84,38.3 |
| F2 | -32 | -119.8,55.8 | 0.47 | 0.93 | -23 | -86.1,40.1 |
| APOD | -47.2 | -192,97.7 | 0.52 | 0.93 | -21.9 | -89.1,45.3 |
| APOM | -42.5 | -180.5,95.5 | 0.54 | 0.93 | -20 | -84.9,44.9 |
| IGKC | -20.3 | -98.3,57.7 | 0.6 | 0.93 | -16.5 | -79.7,46.8 |
| ALB | 51.4 | -146.7,249.6 | 0.6 | 0.93 | 17 | -48.6,82.7 |
| APOB | 32.2 | -94.2,158.5 | 0.61 | 0.93 | 17 | -49.9,83.9 |
| APOC1 | 15.7 | -56.9,88.4 | 0.66 | 0.96 | 14.7 | -53,82.3 |
| PON1 | 12.3 | -98.4,123 | 0.82 | 0.97 | 7 | -56,70 |
| FGA | 11.3 | -97.1,119.8 | 0.83 | 0.97 | 6.7 | -57.6,71 |
| PPBP | -7 | -84,69.9 | 0.85 | 0.97 | -6.8 | -80.8,67.3 |
| CLU | -18.4 | -227.8,191 | 0.86 | 0.97 | -6 | -74,62.1 |
| VTN | -10.3 | -164.5,143.9 | 0.89 | 0.97 | -4.7 | -75.3,65.8 |
| APOL1 | 6 | -125.9,137.8 | 0.93 | 0.97 | 3 | -62.6,68.5 |
| FGG | -3.7 | -124.1,116.8 | 0.95 | 0.97 | -2 | -67.9,63.9 |
| APOC4 | 1.7 | -59.7,63 | 0.96 | 0.97 | 1.9 | -69.1,73 |
| HP | 2.1 | -109.5,113.7 | 0.97 | 0.97 | 1.3 | -65.6,68.1 |
